# Real‐World National Databases Versus Hypothesis‐Driven Cohort Studies: A Reply to the Critique on the TURK‐HF Registry Design

**DOI:** 10.1002/clc.70385

**Published:** 2026-07-04

**Authors:** Umut Kocabaş

**Affiliations:** ^1^ Department of Cardiology Başkent University Izmir Hospital Izmir Turkey

To the Editor,

We thank Çağrı Zorlu, MD, for his comments concerning the design and methodology of the Türkiye Heart Failure (TURK‐HF) Registry and are grateful for the opportunity to elucidate some key methodological features of the database [1, 2].

We believe that some of the issues raised can be attributed to differences in thinking about the methodological gap between a national registry infrastructure and a hypothesis‐driven registry‐based cohort study. The European Medicines Agency (EMA) has stated that there are relevant methodological differences between registry databases and registry‐based studies [3]. A patient registry is a well‐organized system for collecting uniform data to evaluate specified outcomes for a population defined by a particular disease, condition, or exposure. Registry‐based studies use the infrastructure, patient population, or data generated from these registries to answer specific research questions. Therefore, although patient registry databases, such as the TURK‐HF Registry, collect standardized data according to predefined protocols, coding systems, and operational procedures, hypothesis‐driven registry‐based cohort studies are usually constructed around specific research questions, predetermined confounders, and effect modifiers with dedicated statistical assumptions. Therefore, estimates of sample size, power calculations, and analysis plans should be primarily viewed in the context of analyses to be performed subsequent to registry enrollment and not in the context of the registry infrastructure [3].

Bias and representativeness considerations: Bias is a key issue in any multicenter observational study, and yet, its presence does not preclude a prospective study. However, representativeness, as referred to in registry science, is not synonymous with population‐based random sampling. The selection of the centers that participated followed the principles of the Nomenclature of Territorial Units for Statistics and the projections of the Health Statistics Yearbook; this selection reflected geographical spread as well as different types of healthcare providers [4, 5]. Participation in registration is a voluntary matter, and the mandatory spell of a “complete” registration of all relevant persons, as in the case method, should not be anticipated. Modern cardiovascular registries are largely based on the unsolicited contributions of centers, as mandatory inclusion is almost never feasible in large‐scale, real‐life endeavors. Therefore, it should not be assumed that voluntary enrollment undermines the validity of the registry methodology; rather, it represents a reflection of pragmatic requirements for the prospective collection of nationwide data.

Data quality and reliability: The authors recognize that heterogeneity among the participating sites is an unavoidable challenge in multicenter registries. Thus, data collection was harmonized through a centralized electronic case report form with a standard set of variables and structured data entry, different levels of access, pseudonymized data storage, and predefined operational definitions for the variables to be collected.

Assessment of echocardiographic data: The echocardiographic data were obtained within the confines of routine clinical practice to maintain the real‐world character of the registry; however, predefined echocardiographic variables and standardized definitions were implemented in the electronic case‐report form to enhance uniformity among centers. Hence, the lack of a central core laboratory should be viewed in the context of the intention to achieve methodological standardization and ensure national feasibility and wide dissemination.

The Clinical Frailty Scale of the Canadian Study of Health and Aging was selected for the assessment of frailty due to its multi‐dimensional clinical relevance and its established use within heart failure cohorts [6, 7]. We recognize that physician‐assessed clinical tools are subject to inter‐rater variability, which cannot be completely eradicated in nationwide registries. However, to minimize variability between centers, consistent uniform variable definitions were introduced, and data collection was standardized electronically. Importantly, the intention of the registry was not to alter the variability in clinical practice but to define it systematically, as seen in daily clinical care.

Change over time in the uptake of GDMT, subgroup analyses, and aims of the registry: We consider that this underlines once more the difference between registry infrastructure and analyses based on registries. The TURK‐HF registry database was not intended to be limited to one‐time analyses as treatments change over time. Therefore, changes in treatment over time should not be considered as threats to methodology, but as natural phenomena that the registry tries to capture in a prospective manner through repeated patient visits and reassessments of treatment effects. Similarly, subgroup analyses according to region, type of healthcare provider, or patient phenotype should be interpreted as future analyses within the registry rather than pre‐specified objectives of the registry's infrastructure per se. The power considerations, sampling strategies, and analytical approaches for these analyses will depend on the hypotheses specified for that study.

Socioeconomic status and H2FPEF score: The Boratav framework was applied not to substitute conflicting traditional socioeconomic measures or to increase cross‐national comparability but to increase contextual validity by identifying socioeconomic formations potentially unique to Türkiye while also gathering traditional socioeconomic data [8]. Hence, no single frame of reference was meant to take precedence over the other; rather, a national, real‐world registry should offer relevant and complementary socioeconomic descriptions. Similarly, the H2FPEF score was always intended to be used as a decision‐support tool, not as a stand‐alone diagnostic criterion [9]. We are aware of previous studies in the Turkish population that have shown different performances with H2FPEF‐based algorithms. However, such heterogeneity supports the idea of introducing such tools into prospective national registries, as the registry setup would allow for the assessment of their external validity, calibration, and performance in real life across wider and more varied populations. In this line, the registry was purposely designed to test the applicability of such instruments to real‐world patients rather than wielding them as isolated diagnostic tools.

Clinical end‐point assessment: We agree that independent end‐point adjudication is an important methodological strength in prospective cardiovascular studies and that it may decrease the risk of misclassification of outcomes. However, the lack of centralized end‐point adjudication needs to be interpreted within the limitations of the prospective observational registry design, as feasibility, operational practicality, and securing wide national participation often entail a compromise between methodological rigor and implementation in real life. While it is recognized that the absence of independent adjudication may result in greater vulnerability to outcome misclassification, this limitation needs to be balanced with the goal of developing a national registry infrastructure that can be expanded and used to capture routine clinical practice in a variety of clinical environments.

Guideline‐directed medical therapy and barriers to its implementation: Separating the consideration of barriers to implementation issues from intervention in clinical decision‐making. We believe that it is important to separate the evaluation of aspects of implementation barriers related to clinical decision‐making from active intervention. Investigating barriers to the implementation of guidelines is not inherently incompatible with leaving treatment decisions to the clinical judgment of the participating physicians. The TURK‐HF Registry was not designed to promote or influence clinical care or harmonize treatment decisions or therapeutic options for patients; it was primarily initiated to reflect real‐world practice patterns and to define the barriers hampering the application of evidence‐based therapy, according to our aim. Importance of distinguishing Implementation Science and Implementation Assessment from Implementation itself. Failure to carry out the implemented program or activity of intervention properly: known as a type III error. Knowing why evidence‐based therapies are not prescribed, started, up‐titrated, or maintained is essential for formulating intervention strategies that may enhance delivery of care. To this end, we added a structured treatment evaluation algorithm to an electronic case report form. This is a reporting—rather than a modeling—algorithm describing treatment use, molecule choice, dose density, and patient‐ and physician‐related causes of drug non‐use. We acknowledge that self‐reported rationales may be subject to reporting biases, such as social desirability bias. Nevertheless, the prospective documentation of these items certainly adds more details than the simple reporting of medication use rates. This process ensures that future registry‐based investigations can address open questions regarding the translation into day‐to‐day clinical routines. Importantly, the participating investigators were free to initiate, optimize, and provide long‐term follow‐up management of therapy. The purpose of the registry infrastructure was to monitor routine care and not to influence it.

In summary, although registry‐based studies are hypothesis‐driven, they are not designed with predetermined effect sizes, nor do they employ dedicated sampling strategies, as this is expected from a registry database, the primary purpose of which is to provide a broad prospective real‐world building block with the capability to support a multitude of future analyses. Accordingly, the TURK‐HF Registry aims not to form a hypothesis‐driven analytic cohort for a single prespecified research question but to establish the infrastructure of a national registry that reflects the diverse spectrum of HF care in Türkiye.

## Conflicts of Interest

Umut Kocabaş has received speaker's honoraria for lectures at meetings sponsored by Daiichi‐Sankyo, Bayer, Abdi İbrahim, Sanovel, and Santa Farma drug companies and has received a consulting fee from the GlaxoSmithKline drug company.

## Data Availability

The data that support the findings of this study are available on request from the corresponding author. The data are not publicly available due to privacy or ethical restrictions.
